# Snail accelerates cancer invasion by upregulating MMP expression and is associated with poor prognosis of hepatocellular carcinoma

**DOI:** 10.1038/sj.bjc.6602266

**Published:** 2005-01-25

**Authors:** A Miyoshi, Y Kitajima, S Kido, T Shimonishi, S Matsuyama, K Kitahara, K Miyazaki

**Affiliations:** 1 Department of Surgery, Saga University Faculty of Medicine, 5-1-1 Nabeshima, Saga 849-8501, Japan

**Keywords:** hepatocellular carcinoma, Snail, matrix metalloproteinase, cancer invasion

## Abstract

We have previously demonstrated in an *in vitro* study that Snail increased the invasion activity of hepatoma cells by upregulating matrix metalloproteinase (MMP) gene expression. In the present study, we examined whether Snail gene expression correlates with cancer invasion and prognosis of patients with hepatocellular carcinoma (HCC). Quantitative reverse transcription–polymerase chain reaction (RT–PCR) was performed to evaluate Snail, E-cadherin, and MMP mRNA expressions in eight nodule-in-nodule tumours and 47 ordinary HCC tissues. In the nodule-in-nodule tumours, Snail expression significantly increased with tumour dedifferentiation (*P*=0.047). In the ordinary HCC tissues, Snail expression was significantly correlated with portal vein invasion (*P*=0.035) and intrahepatic metastasis (*P*=0.050); it also showed a significant correlation with MT1-MMP expression (*r*=0.572, *P*<0.001). In recurrence-free survival, the group with high Snail expression showed significantly poorer prognosis (*P*=0.035). Moreover, high Snail expression was an independent risk factor for early recurrence after curative resection. During the progression of HCC, Snail expression may be induced and accelerate invasion activity by upregulating MMP expression, resulting in portal invasion, intrahepatic metastasis, and poor prognosis.

Hepatocellular carcinoma (HCC) is one of the most prevalent malignancies in the world and a frequent cause of cancer fatalities in Japan. Improvements in early diagnosis, surgical techniques, and perioperative management have contributed to decreases in mortality and morbidity among patients with HCC (Okuda *et al*, 1985; Fran *et al*, 1999). However, the long-term prognosis of patients with HCC after hepatectomy has been still poor because of a high incidence of recurrence after initial treatment (Fong *et al*, 1999; Poon *et al*, 2000b). Several centres have reported a cumulative 5-year recurrence rate ranging from 75 to 100% (Chen *et al*, 1994; Fong *et al*, 1999; Poon *et al*, 2000b). Pathological and genetic analyses have indicated two features in HCC recurrence: multicentric occurrence of new tumours (MO) and intrahepatic metastasis of the original tumour (im) (Tsuda *et al*, 1992; Matsumoto *et al*, 2001). It has been reported that MO is significantly influenced by the underlying liver status, such as the presence of active hepatitis (Belghiti *et al*, 1991; Ko *et al*, 1996). On the other hand, im is thought to be more closely associated with tumour factors, especially portal vein invasion (vp) (Vauthey *et al*, 1995; Shimada *et al*, 1999). Several published studies have demonstrated that vp plays an important role in the im process and influences the survival rate of patients with HCC (Fuster *et al*, 1996; Mitsunobu *et al*, 1996; The Liver Cancer Study Group of Japan, 1994). Therefore, it is important to elucidate the molecular mechanisms of vp and im; the subsequent establishment of markers for predicting vp and im may contribute to improvement in the prognosis of patients with HCC.

Recently, the zinc-finger transcription factor Snail has been reported to repress E-cadherin expression, which mediates cell–cell adhesion, and to increase cancer invasion in various malignancies (Battle *et al*, 2000; Yokoyama *et al*, 2001). We have demonstrated in an *in vitro* study using HCC cell lines that Snail not only represses E-cadherin but also increases gene expression of the matrix metalloproteinase (MMP) family, which is thought to accelerate cancer invasion (Miyoshi *et al*, 2004).

In this study, we attempted to clarify the *in vivo* significance of Snail expression in HCC progression. We evaluated Snail, E-cadherin, and MMP gene expressions in surgically resected HCC tissues by quantitative reverse transcription–polymerase chain reaction (RT–PCR). Subsequently, we assessed the relationship between Snail gene expressions and those of E-cadherin and MMP. Snail expression was further compared with the clinicopathologic factors and prognosis of patients with HCC.

## MATERIALS AND METHODS

### Patients

From January 1998 to January 2003, 95 patients with primary HCC consecutively underwent curative resection in the Department of Surgery, Saga University Faculty of Medicine. Curative resection was defined as complete excision of the tumour with clear microscopic margins and no residual tumours demonstrated by computed tomography (CT) scanning. Of these 95 patients, 10 patients who underwent preoperative transarterial embolisation were excluded from this study. Another 30 patients were excluded because their RNA samples degraded.

**Figure 1:**
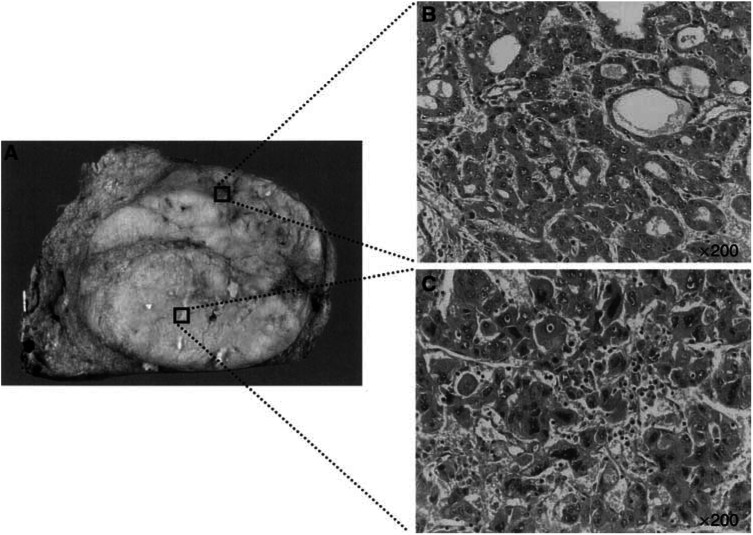
Macroscopic and microscopic features of HCC showing a NIN appearance. (**A**) Macroscopically, the solitary tumour was separated into an outer nodule and an inner nodule by a septum. (**B**) Microscopically, the outer nodule showed a moderately differentiated HCC, forming in a pseudoglandular fashion. (**C**) Hepatocellular carcinoma cells in the inner nodule were poorly differentiated with large hyperchromatic nuclei. Microscopic photographs were taken at a magnification of × 200.

Among the 55 patients remaining in the study, eight patients had tumours with a nodule-in-nodule appearance (NIN) on macroscopic and microscopic findings (Figure 1). Hepatocellular carcinoma with NIN was better tissue for investigating the dedifferentiation and progression of HCC because the inner nodule of less differentiation developed sequentially from the well-differentiated outer nodule with the same genetic background (Kojiro, 1998; Midorikawa *et al*, 2002).

Eight patients with NIN and 47 patients without NIN were the subjects of this study and these data sets were analysed separately.

Written informed consent that was recognised by the ethical committee in Saga University Faculty of Medicine was obtained from each patient before tissue acquisition. The eight patients with NIN were six men and two women with a mean age of 68.7 years (range, 60–74 years). The mean tumour size was 50.1 mm (range, 17–110 mm). Histologically, four tumours had well-differentiated HCC in the outer nodules and moderately differentiated HCC in the inner nodules; the other four tumours had moderately differentiated HCC in the outer nodules and poorly differentiated HCC in the inner nodules.

The 47 patients without NIN were 33 men and 14 women with a mean age of 65.7 years (range, 48–84 years). Four patients (8.5%) were positive for the hepatitis B surface antigen and 39 (83.0%) were positive for the hepatitis C antibody. Four patients (8.5%) were negative for both of these viruses. A total of 24 patients (51.1%) had liver cirrhosis. The mean tumour size was 45.7 mm (range, 13–170 mm). The histological grade of each tumour was determined according to the General Rules for the Clinical and Pathological Study of Primary Liver Cancer (The Liver Cancer Study Group of Japan, 2000). In all, 21 tumours (44.7%) were well-differentiated HCC and 26 (55.3%) were moderately differentiated HCC. Of these 47 patients, 42 underwent anatomical resections according to Couinaud's segments (Couinaud, 1957). The other five patients underwent nonanatomical resections, including limited resection and tumour enucleation. All 47 patients received follow-up examinations of tumour markers, ultrasonography (US), and CT every 3 months. The median follow-up period was 42 months (range, 12–69 months). In this period, tumour recurrence was observed in 24 patients (51.1%) and early recurrence (<1 year) was observed in 11 patients (23.4%). The liver was the first site of recurrence in all of these cases.

### RNA extraction

In the eight HCC frozen tissues with NIN, total RNA was extracted from both the inner and outer nodules in 30 mg frozen tissue using RNeasy™ Mini Kits (Qiagen, Hilden, Germany) according to the manufacturer's protocol. In the 47 HCC tissues without NIN, total RNA was extracted from both the cancer and noncancerous liver tissues using the same protocol. All RNA samples were confirmed to have no degradation by electrophoresis on 1% agarose gel.

### Quantitative RT–PCR

The RNA samples (1 *μ*g) were converted into cDNA by reverse transcription (RT) using random primers (TAKARA, Siga, Japan) according to the manufacturer's instructions. To quantitatively estimate the mRNA expressions of several genes, polymerase chain reaction (PCR) amplification was performed on a Light-Cycler™ instrument system (Roche, Mannheim, Germany) using the Light-Cycler-FastStart™ DNA Master SYBR green I kit (Roche). Amplifications were performed in a 20-*μ*l solution of 4 mM MgCl_2_, 20 pmol of primer, 2 *μ*l of Light-Cycler-FastStart DNA Master SYBR green I reagent, and 2 *μ*l of cDNA. After 3 min denaturation at 95°C, amplifications were carried out with 50 cycles of 15 s denaturation at 95°C, 5 s annealing at 60°C, and 10 s extension at 72°C. Melting curves were obtained according to the protocol under the following conditions: 0 s denaturation period at 95°C, starting temperature of 65°C, ending temperature of 95°C, and rate of temperature increase of 0.1°C s^−1^. The sequences of the PCR primer pair and the fragment size are shown in Table 1. These experiments were carried out in triplicate.

### Quantification of expression

Relative expression levels of target genes were determined from standard curves using the control plasmid pSTBluel (Novagen, Madison, WI, USA) inserted into the cDNA fragments of E-cadherin, Snail, or MMP genes, and serially diluted to 3 pg ml^−1^, 30 pg ml^−1^, 300 pg ml^−1^, and 3 ng ml^−1^. The quantitative value of the target gene in each sample was normalised using GAPDH expression as an internal control. The quantitative RT–PCR assay was carried out in triplicate and the mean value was calculated. Finally, the mRNA expression ratio of cancerous (C) to noncancerous (N) tissues was calculated from the following formula: *R*={target gene (C)/GAPDH (C)}/{target gene (N)/GAPDH (N)}. High Snail expression was defined as *R*>1.0 and low Snail expression as *R*⩽1.0.

### Statistical analysis

Statistical analysis was performed using the StatView™ version 5.0 software system (SAS Insititute Inc., Cary, NC, USA). On analysis of the samples with NIN, gene expressions were compared between well-differentiated and less-differentiated nodules using the paired Student's *t*-test. A Pearson's correlation test was used to assess the correlation between Snail and MMP family gene expressions in which each value of mRNA expression was estimated as log transformed before statistical analysis. The relationships between Snail expression levels and clinicopathologic features were evaluated using the unpaired Student's *t*-test. Survival rates were calculated by the Kaplan–Meier methods and compared by the Wilcoxon test. To elucidate the risk factors for early recurrence (< 1 year), univariate analysis was performed using the *χ*^2^ test and multivariate analysis was carried out using the logistic regression model. A *P*-value of less than 0.05 was considered to be statistically significant.

## RESULTS

### Expressions of Snail and MMP family genes in HCC tissues with NIN

**Figure 2:**
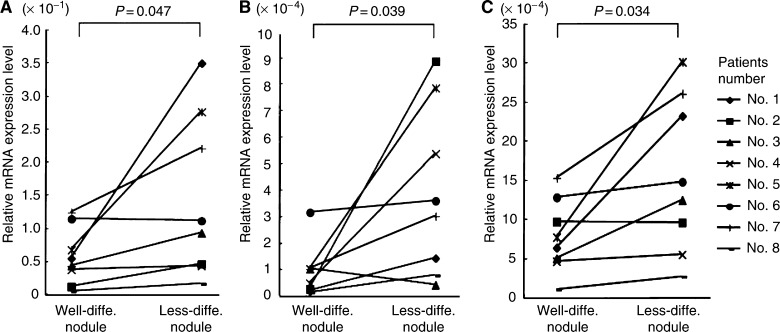
Comparison of Snail (**A**), MMP-7 (**B**), and MTl-MMP (**C**) expressions between well-differentiated and less-differentiated nodules (*n*=8). Relative mRNA levels of these genes were significantly increased in the less-differentiated nodules. Statistical analyses were carried out using a paired Student's *t*-test.

**Figure 3:**
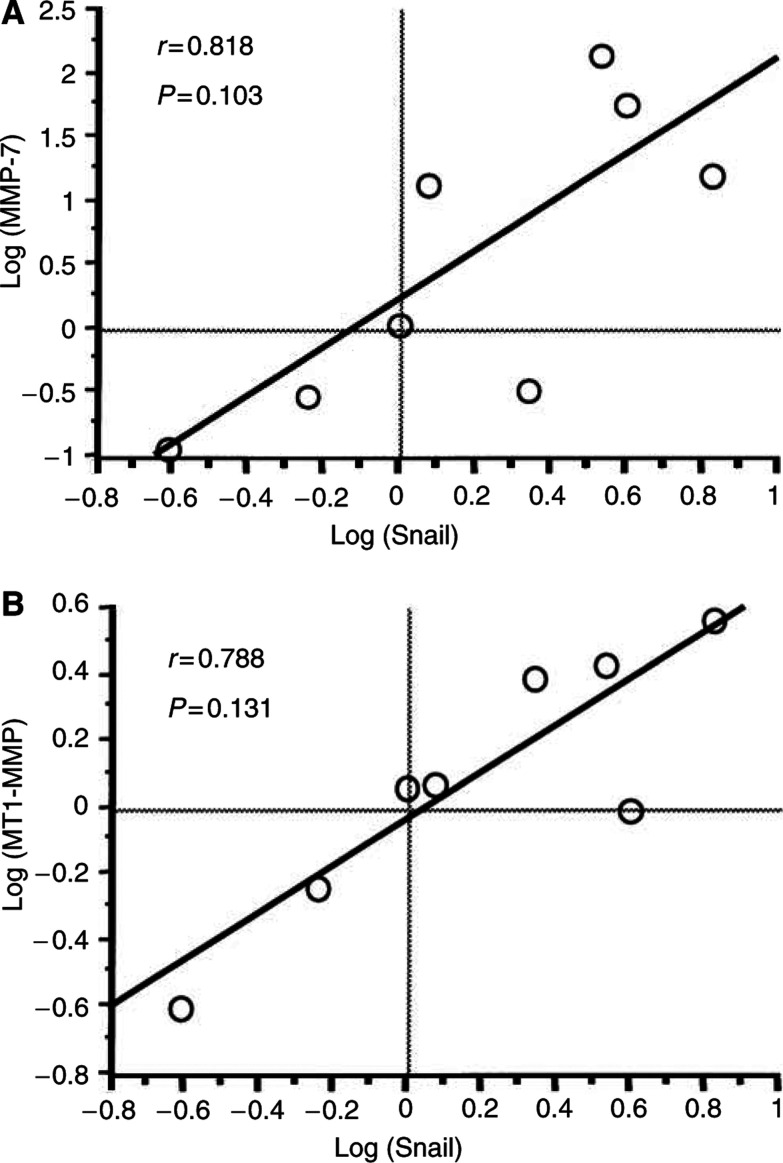
Correlation between Snail expression and MMP expression in tumours with a NIN appearance (*n*=8). (**A**) A positive correlation was found between Snail expression and MMP-7 expression (*r*=0.818, *P*=0.103). (**B**) A positive correlation was found between Snail expression and MT1-MMP expression (*r*=0.788, *P*=0.131). All data were log transformed before statistical analysis.

Using HCC tissues with NIN, we quantitatively analysed the gene expressions of Snail, E-cadherin, and the MMP family in the outer as well as the inner nodules of NIN tumours and compared the results with the differentiation grades. As shown in Figure 2, Snail expression was significantly higher in the less-differentiated nodules than in the well-differentiated nodules (*P*=0.047) (Figure 2A). In less-differentiated nodules, the expression of MMP-7 was significantly increased (*P*=0.039) (Figure 2B) as well as the expression of MT1-MMP (*P*=0.034) (Figure 2C). However, MMP-2 expression did not significantly vary with differentiation (data not shown). The MMP-1 mRNA was not detectable in most of the tissues (data not shown). Figure 3 shows the correlation between Snail expression and MMP expression. Snail expression had a trend correlating with MMP-7 expression (*r*=0.818) (Figure 3A) and MT1-MMP expression (*r*=0.788) (Figure 3B). However, these correlations were not significant because of the small number of samples examined. No correlation was found between E-cadherin expression and Snail expression in HCC with NIN (data not shown).

### Relationship between Snail expression and clinicopathologic features in 47 patients with ordinary HCC

To elucidate the biologic significance of Snail expression in HCC, we correlated the Snail expression with the clinicopathologic features of 47 HCC tissue samples. As shown in Table 2, the mean amount of Snail expression was significantly higher in HCC tumours with vp (*P*=0.035) and im (*P*=0.050) than in tumours without these parameters. However, Snail expression was not correlated with histological grade.

### Correlation between Snail expression and MMP expression in 47 patients with ordinary HCC

**Figure 4:**
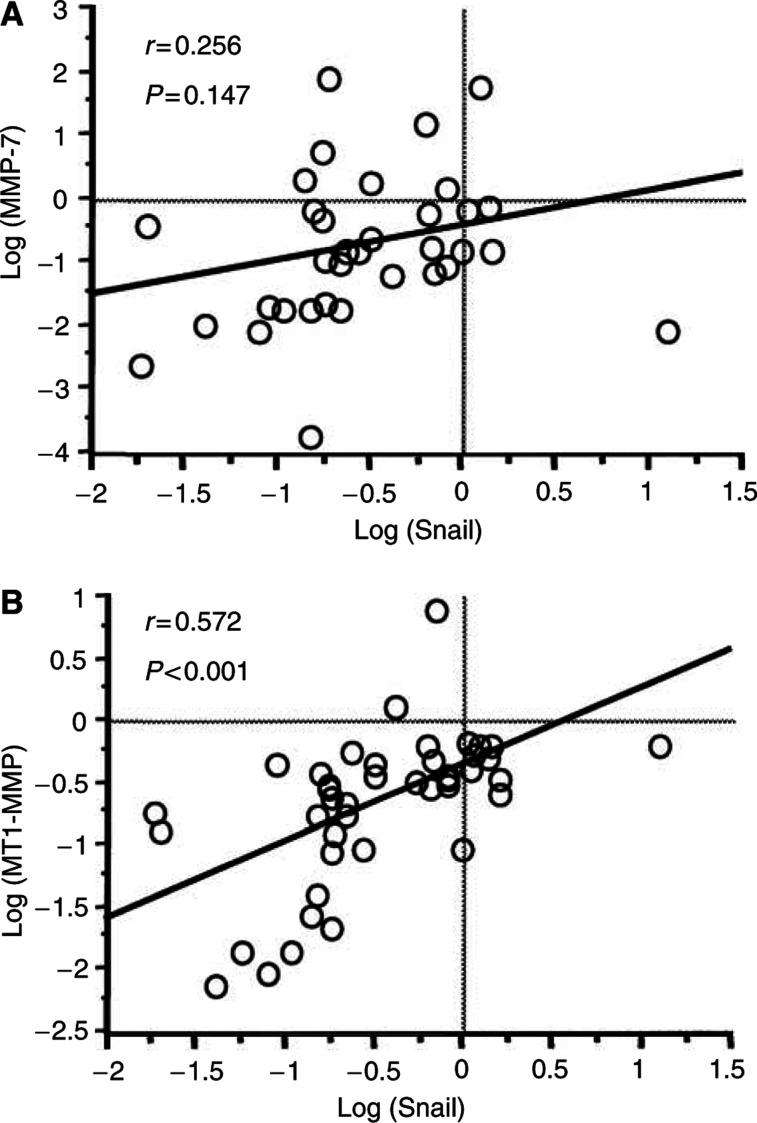
Correlation between Snail expression and MMP expression in 47 consecutive tumours. (**A**) A positive correlation was found between Snail expression and MMP-7 expression (*r*=0.256, *P*=0.147), but it was not significant. (**B**) A significant positive correlation was found between Snail expression and MT1-MMP expression (*r*=0.572, *P*<0.001). All data were log transformed before statistical analysis.

Figure 4 shows the correlation between mean amounts of Snail expression and MMP expression in 47 HCC tissue samples. A positive correlation was found between Snail expression and MMP-7 expressions (*r*=0.256, *P*=0.147); however, the correlation was not strong (Figure 4A). A strongly positive correlation was found between Snail expression and MT1-MMP expression (*r*=0.572, *P*<0.001) (Figure 4B). E-cadherin expression was not correlated with Snail expression (data not shown).

### Prognostic significance of high Snail expression

**Figure 5:**
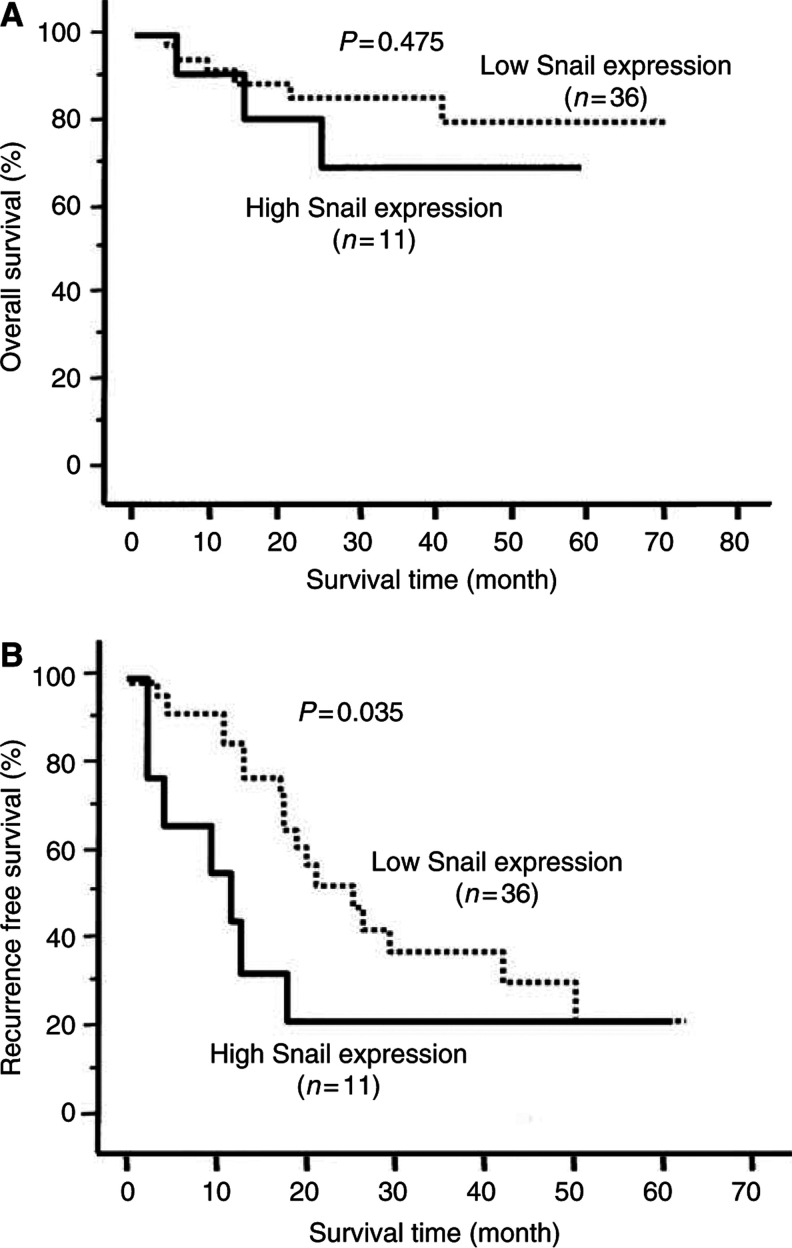
Kaplan–Meier curves of 47 patients with HCC after curative resection. (**A**) Regarding the overall survival rate, no significant difference was found between the group with high Snail expression and the group with low Snail expression (*P*=0.475; Wilcoxon test). (**B**) Regarding the recurrence-free survival rate, the group with high Snail expression (*n*=11) had a significantly poorer prognosis compared with the group with low Snail expression (*n*=36) (*P*=0.035; Wilcoxon test).

Overall and recurrence-free survival rates were estimated by Kaplan–Meier curves (Figure 5). Of 47 patients, 11 (23.4%) with HCC had high Snail expression and 36 (76.6%) had low Snail expression. Regarding overall survival, Snail expression was not significantly associated with the prognosis of patients with HCC (Figure 5A). However, regarding recurrence-free survival, the group with high Snail expression showed significantly poorer prognosis than the group with low Snail expression (Figure 5B) (*P*=0.035). In particular, 54.5% of patients in the high Snail expression group developed a metastasis within 12 months after curative resection compared with 13.8% of patients in the low Snail expression group. On the other hand, the patients in the low Snail expression group had an increased rate of recurrence after 12 months.

### Risk factors for early tumour recurrence

Among the 47 patients, 24 patients (51.1%) experienced tumour recurrence and 11 (23.4%) experienced early recurrence (within 1 year). As shown in Table 3, early recurrence was compared with various factors including Snail expression. Six of the 11 patients with high Snail expression and five of the 36 patients with low Snail expression relapsed within 12 months. As a result of statistical analysis using the *χ*^2^ test, high Snail expression showed significant correlation with early tumour recurrence (*P*=0.005). Alpha-fetoprotein (*P*=0.153) and histological grade (*P*=0.148) were also associated with early tumour recurrence. Multivariate analysis was further carried out using logistic regression analysis (Table 4). The analysis demonstrated that high Snail expression was an independent risk factor for early recurrence of HCC after hepatic resection (risk ratio 10.174, *P*=0.015).

## DISCUSSION

Zinc-finger transcription factor Snail has been isolated in Drosophila embryos (Grau *et al*, 1984). During the embryonic development, Snail has been implicated in the triggering of the epithelial–mesenchymal transition (EMT) in the precursors of the mesoderm and neural crest (Cano *et al*, 2000). Snail is known to directly repress E-cadherin gene transcription by binding to the E-box on the E-cadherin promoter (Batlle *et al*, 2000). Several reports have also implicated Snail not only in E-cadherin repression but also in the acceleration of cancer invasion in various carcinomas (Batlle *et al*, 2000; Yokoyama *et al*, 2001). Blanco *et al* (2002) have reported that Snail expression correlated with histological grade and lymph node status in breast carcinomas, which was demonstrated by the *in situ* hybridisation technique. Sugimachi *et al* (2003) reported in a study using real-time RT–PCR that Snail mRNA levels independently correlated with capsular invasion in HCC tissues. As reported above, RT–PCR or *in situ* hybridisation has been performed to assess Snail expression because available Snail antibody has not been isolated. We have also reported the results of an *in vitro* study concerning Snail expression in HCC cells. First, we demonstrated an inverse correlation between Snail and E-cadherin expression in various HCC cells in which differentiated HCC cells expressed E-cadherin but not Snail and undifferentiated HCC cells expressed Snail but not E-cadherin (Jiao *et al*, 2002). Then, we established Snail transfectants in differentiated hepatoma HepG2 cells and evaluated the biologic alteration by Snail introduction. As a result, we found that E-cadherin expression was repressed in the Snail transfectant, furthermore, EMT was dramatically induced and invasion activity was increased about 10-fold compared with that of mock transfectant cells. We further discovered that MMP-1, MMP-2, MMP-7, and MT1-MMP expressions were significantly upregulated by Snail introduction (Miyoshi *et al*, 2004). The MMP family is known to play a key role in tumour invasion of various human cancers including HCC (Yamamoto *et al*, 1997; Harada *et al*, 1998; Nabeshima *et al*, 2002). Increased expression of MMP-2, MMP-7, and MT1-MMP had a strong association with dedifferentiation, portal invasion, intrahepatic metastasis, and recurrence in HCC (Yamamoto *et al*, 1997; Harada *et al*, 1998). In particular, MT1-MMP appeared to be the most important factor in HCC progression because of its widespread pattern of expression (Harada *et al*, 1998).

On the basis of these findings, we hypothesised that Snail represses E-cadherin expression, which may lead to HCC dedifferentiation, and induces MMP expression, which causes vascular invasion and intrahepatic metastasis in the primary tumour of HCC.

In the present study, we first analysed eight HCC tissues with NIN tissues that were thought to express typical features of dedifferentiation and progression processes in HCC. We examined the quantitative expressions of Snail, E-cadherin, and MMP mRNAs in eight HCC tissues with NIN. As a result, Snail expression significantly increased in the less differentiated nodules, compared with the well-differentiated nodules. Snail expression had a trend of correlation with MMP-7 and MT1-MMP expressions. These results strongly suggest that Snail was induced during HCC dedifferentiation and the gene product upregulated MMP-7 and MT1-MMP expressions. We further investigated the relationship between Snail expression and clinicopathologic features in 47 patients with HCC who underwent curative resection. We provided evidence that Snail expression was significantly associated with vp and im. In the 47 HCC tissues, there was a significant correlation between Snail and MT1-MMP expression. These results implied that Snail accelerated cancer invasion via upregulating MMP gene, especially MT1-MMP, leading to the development of vp and im in HCC. However, Snail expression did not correlate with histological grade in these 47 HCC specimens, unlike the finding in eight samples of HCC with NIN in which Snail was induced with tumour dedifferentiation. HCC tumours sometimes have microscopically heterogenous differentiation. In the general rules for the clinical and pathological study of primary liver cancer, tumour grade was decided according to the majority. The 47 ordinary HCC samples may have cancer contents with heterogenous differentiation and the differentiation state of frozen specimens may not always be consistent with the corresponding histological grade. In addition, an inverse correlation between E-cadherin expression and Snail expression had been found in a previous study using HCC cell lines; however, no association was found between the two gene expressions in the 47 HCC and eight NIN tissue samples in this study. Several reports have shown two mechanisms in the downregulation of E-cadherin in HCC; one is the hypermethylation of the E-cadherin promoter and the other is the loss of heterozygosity of the E-cadherin gene (Kanai *et al*, 1997; Matsumura *et al*, 2001; Wei *et al*, 2002). The authors demonstrated that DNA hypermethylation of the E-cadherin promoter region was observed in 33–67% and loss of heterozygosity (LOH) was detected in 30–43% of HCC tissues. It was reported that loss of E-cadherin expression was associated with LOH of E-cadherin locus and methylation in the promoter region. Sugimachi *et al* (2003) reported that Snail overexpression was observed in 16% of HCC patients and there were 69% with reduced E-cadherin expression but without Snail overexpression. In the present study, Snail overexpression was detected in 11(23%) of 47 HCC patients but an inverse correlation between Snail and E-cadherin was not observed. Although the apparent reason remains uncertain, Snail might play a lesser role in E-cadherin repression than those of promoter hypermethylation and LOH. Finally, regarding the recurrent-free survival rate, the high Snail expression group showed significantly poorer prognosis than that with low Snail expression. In particular, six of 11 (54.5%) patients with high Snail expression experienced tumour recurrence within 1 year after hepatic resection. On the other hand, the incidence of tumour recurrence of patients with low Snail expression was increased during 1 year after hepatectomy. Several reports have indicated that early recurrence (<1 year) after hepatic resection arose mainly from im and resulted in a significantly worse prognosis than late recurrence (>1 year). Conversely, late recurrence was reported to occur most likely due to MO (Jwo *et al*, 1992; Poon *et al*, 2000a; Imamura *et al*, 2003). Therefore, it is suggested that tumour recurrence in the high Snail expression group is associated with im, which possibly spread through the portal vein invaded by the primary tumour. To identify risk factors affecting early tumour recurrence (<1 year), we performed multivariate analysis as well as univariate analysis with clinicopathologic parameters that were reported to be associated with the tumour recurrence (Chen *et al*, 1994; Fong *et al*, 1999; Poon *et al*, 2000a). As a result, high Snail expression was revealed as an independent risk factor for early recurrence (<1 year). Several reports showed that vp was associated with early recurrence (Fong *et al*, 1999; Shimada *et al*, 1999; Poon *et al*, 2000a). However, in the present study, vp did not correlate with early recurrence. We analysed 178 patients with HCC who underwent hepatic resection from 1984 to 2003. As a result of the analysis, vp correlated with early tumour recurrence (not published data). So, it is supposed that small numbers affect this difference in the present study.

In conclusion, we elucidated a novel invasion mechanism of HCC that is triggered by Snail gene expression. Clinically, Snail may be a crucial marker for predicting vp, im, and early recurrence after hepatic resection.

## Figures and Tables

**Table 1:** Sequence of PCR primer and fragment size

**Gene**	**Sequence**	**Fragment size (bp)**
E-cadherin	5′-TCGACACCCGATTCAAAGTGG-3′	194
	3′-TTCCAGAAACGGAGGCCTGAT-5′	
Snail	5′-TATGCTGCCTTCCCAGGCTTG-3′	145
	3′-ATGTGCATCTTGAGGGCACCC-5′	
MMP-1	5′-GATGTTCAGCTAGCTCAGGAT-3′	193
	3′-AAGGGATTTGTGCGCATGTAG-5′	
MMP-2	5′-CTTCTTCAAGGACCGGTTCAT-3′	183
	3′-GCTGGCTGAGTAGATCCAGTA-5′	
MMP-7	5′-ATGGGGAACTGCTGACATCAT-3′	153
	3′-CCAGCGTTCATCCTCATCGAA-5′	
MT1-MMP	5′-CTAAGACCTTGGGAGGAAAAC-3′	192
	3′-AAGCCCCATCCAAGGCTAACA-5′	
GAPDH	5′-GGCGTTTTGCAATGCAGATGTAG-3′	189
	3′-CACAGGAGCCGTCACTTCTCTTG-5′	

**Table 2:** Relationship between Snail mRNA expression and clinicopathologic features

	**Snail mRNA expression**		
**Factors**	***N***	**(Mean±s.d.)**	***P*-value**
*Gender*			
Male	33	0.841±2.084	
Female	14	1.132±2.170	0.668
*Age (years)*			
⩽65	19	1.128±2.720	
>65	28	0.792±1.570	0.594
*Liver cirrhosis*			
Absent	23	0.862±1.718	
Present	24	0.991±2.430	0.835
*Alpha-fetoprotein (ng ml^−1^)*			
⩽10	12	0.578±0.488	
>10	35	1.047±2.403	0.508
*Tumour multiplicity*			
Solitary	28	0.556±0.517	
Multiple	19	1.476±3.199	0.140
*Tumour size (mm)*			
⩽50	33	0.792±2.084	
>50	14	1.248±2.147	0.500
*Histological grade*			
Well	21	1.165±2.582	
Moderate	26	0.736±1.619	0.490
*Capsular invasion (fc-inf)*			
Absent	14	0.281±0.268	
Present	31	1.258±2.516	0.157
*Portal vein invasion (vp)*			
Absent	35	0.556±0.486	
Present	12	2.011±3.978	0.035
*Hepatic vein invasion (vv)*			
Absent	36	0.751±1.403	
Present	11	1.506±3.568	0.299
*Intrahepatic metastasis (im)*			
Absent	32	0.535±0.509	0.050
Present	15	1.765±3.561	

Well=well-differentiated HCC, Moderate=moderately differentiated HCC, fc-inf, vp, vv, im, diagnosed by pathological findings.

**Table 3:** Univariate analysis for risk factor associated with early tumour recurrence

	**Early recurrence**		
**Factors**	***N***	**(%)**	***P*-value**
*Gender*			
Male	33	8 (24.2)	
Female	14	3 (27.2)	0.839
*Age (years)*			
⩽65	19	3 (15.7)	
>65	28	8 (28.5)	0.309
*Liver cirrhosis*			
Absent	23	6 (26.0)	
Present	24	5 (20.8)	0.670
*Alpha-fetoprotein (ng ml^−1^)*			
⩽10	12	1 (8.3)	
>10	35	10 (28.5)	0.153
*Tumour multiplicity*			
Solitary	28	8 (28.5)	
Multiple	19	3 (15.7)	0.309
*Tumour size (mm)*			
⩽50	33	8 (24.2)	
>50	14	3 (21.4)	0.834
*Histological grade*			
Well	21	4 (15.3)	
Moderate	26	7 (33.3)	0.148
*Capsular invasion (fc-inf)*			
Absent	14	4 (28.5)	
Present	31	7 (22.5)	0.665
*Portal vein invasion (vp)*			
Absent	35	9 (25.7)	
Present	12	2 (16.7)	0.523
*Hepatic vein invasion (vv)*			
Absent	36	9 (25.0)	
Present	11	2(18.2)	0.640
*Intrahepatic metastasis (im)*			
Absent	32	8 (25.0)	
Present	15	3 (20.0)	0.705
*Snail expression level*			
Low	36	5 (13.8)	
High	11	6 (54.5)	0.005

Well=well-differentiated HCC, Moderate=moderately differentiated HCC, fc-inf, vp, vv, im, diagnosed by pathological findings.

**Table 4:** Multivariate logistic regression analysis for risk factor contributing early tumour recurrence

**Variables**	**Risk ratio**	**95% CI**	***P*-value**
Snail expression (high)	10.174	1.561–66.321	0.015
Alpha-fetoprotein (>10 ng ml^−1^)	10.199	0.709–146.787	0.087
Histological grade (moderate)	1.386	0.219–8.757	0.728

CI=confidence interval, Moderate=moderately differentiated HCC.
